# Organic Production Enhances Milk Nutritional Quality by Shifting Fatty Acid Composition: A United States–Wide, 18-Month Study

**DOI:** 10.1371/journal.pone.0082429

**Published:** 2013-12-09

**Authors:** Charles M. Benbrook, Gillian Butler, Maged A. Latif, Carlo Leifert, Donald R. Davis

**Affiliations:** 1 Center for Sustaining Agriculture and Natural Resources, Washington State University, Pullman, Washington, United States of America; 2 School of Agriculture, Food and Rural Development, Newcastle University, Northumberland NE, United Kingdom; 3 Organic Valley/CROPP Cooperative/Organic Prairie, Lafarge, Wisconsin, United States of America; Indiana University, United States of America

## Abstract

Over the last century, intakes of omega-6 (ω-6) fatty acids in Western diets have dramatically increased, while omega-3 (ω-3) intakes have fallen. Resulting ω-6/ω-3 intake ratios have risen to nutritionally undesirable levels, generally 10 to 15, compared to a possible optimal ratio near 2.3. We report results of the first large-scale, nationwide study of fatty acids in U.S. organic and conventional milk. Averaged over 12 months, organic milk contained 25% less ω-6 fatty acids and 62% more ω-3 fatty acids than conventional milk, yielding a 2.5-fold higher ω-6/ω-3 ratio in conventional compared to organic milk (5.77 vs. 2.28). All individual ω-3 fatty acid concentrations were higher in organic milk—α-linolenic acid (by 60%), eicosapentaenoic acid (32%), and docosapentaenoic acid (19%)—as was the concentration of conjugated linoleic acid (18%). We report mostly moderate regional and seasonal variability in milk fatty acid profiles. Hypothetical diets of adult women were modeled to assess milk fatty-acid-driven differences in overall dietary ω-6/ω-3 ratios. Diets varied according to three choices: high instead of moderate dairy consumption; organic vs. conventional dairy products; and reduced vs. typical consumption of ω-6 fatty acids. The three choices together would decrease the ω-6/ω-3 ratio among adult women by ∼80% of the total decrease needed to reach a target ratio of 2.3, with relative impact “switch to low ω-6 foods” > “switch to organic dairy products” ≈ “increase consumption of conventional dairy products.” Based on recommended servings of dairy products and seafoods, dairy products supply far more α-linolenic acid than seafoods, about one-third as much eicosapentaenoic acid, and slightly more docosapentaenoic acid, but negligible docosahexaenoic acid. We conclude that consumers have viable options to reduce average ω-6/ω-3 intake ratios, thereby reducing or eliminating probable risk factors for a wide range of developmental and chronic health problems.

## Introduction

Dairy products contribute significantly to dietary intakes of saturated fat in the United States and Europe, which has led to widely endorsed recommendations to limit consumption of whole milk and other high-fat dairy products, in favor of low- and non-fat dairy products [1]. However, these recommendations are based primarily on the serum-LDL (“bad”)-cholesterol-raising effect of dairy fat, a single marker of risk for cardiovascular disease (CVD). They give little or no consideration to the CVD-risk reducing components in milk fat, especially omega-3 (ω-3) fatty acids (FAs), conjugated linoleic acid (CLA), the possibly beneficial *trans* FAs, *trans*-18:1 [2] and *trans*-16:1 [3], protective minerals, and a beneficial effect on serum HDL (“good”) cholesterol [4].

Two recent reviews of epidemiological evidence question common beliefs about the health effects of dairy fat. One finds a contradiction between the evidence from long-term prospective studies and perceptions of harm from the consumption of dairy products [5]. The other review highlights inconsistent evidence of harm [4]. Most of the reviewed studies began before low-fat dairy products became widely used. These reviews conclude that high consumption of milk and milk fat may be overall neutral [4] or beneficial [5] regarding all-cause mortality, ischemic heart disease, stroke, and diabetes. Most recently, Ludwig and Willett have questioned the scientific basis for recommending reduced-fat dairy products [6]. Additional studies have linked dairy fat consumption to diminished weight gain [7], attenuated markers of metabolic syndrome, including waist circumference [8], and reduced risk of CVD [9] and colorectal cancer [10].

Milk products are good sources of many nutrients, including several of concern in at least some U.S. population cohorts—calcium, potassium, vitamin D (in fortified milk products), vitamin B_12_, and protein [1], [11]. Alpha-linolenic acid (ALA) and other ω-3 FA are also of concern, and are well recognized in milk products [12], but some major reviews do not mention dairy sources [1], [11]. There is increasing evidence that the dietary balance of ω-3 and ω-6 FA is perhaps as important as the dietary proportions of saturated, monounsaturated, and total fat [13], [14].

Health concerns stemming from increasing dietary ω-6/ω-3 ratios have stimulated research on ways to improve the FA profile of common foods, including milk and dairy products [15]–[23]. Changes in dietary FA intakes during the last century have been brought about largely by: (a) increased consumption of major vegetable oils [13], [24], and (b) generally low consumption of oily fish, vegetables, fruits, and beans [25]. Average dietary ratios of these two classes of polyunsaturated FA in the U.S. have increased from about 5 to about 10, with some ratios in excess of 20 [13], [24]–[27]. The ω-6/ω-3 ratio in human breast milk has also increased dramatically in this time period, driven by changes in maternal dietary ratios [28].

Although the optimal ω-6/ω-3 ratio depends on the health measure in question and genetic factors [13], some authors have suggested a target ratio of 2.3. At this ratio, the conversion of ALA to long-chain ω-3 docosahexaenoic acid (DHA) is thought to be maximized [26], [27]. In addition, epidemiological studies have reported no further CVD-prevention benefits from lowering the ω-6/ω-3 ratio below ∼2.3 [24], [25], [29]–[31].

Milk from cows consuming significant amounts of grass and legume-based forages contains higher concentrations of ω-3 FAs and CLA than milk from cows lacking routine access to pasture and fed substantial quantities of grains, especially corn [15]–[23], [32]. In turn, lactating women consuming such milk have an increased CLA concentration in their breast milk [33]. The balance of FAs in animal-derived foods like milk depends on the animal’s dietary lipid intake and on its digestive physiology. The relationship between diet composition and lipid transfer into milk, meat and eggs has been reviewed by Woods and Fearon [34].

The rumen in dairy cattle influences the suitability of different feeds and also has a major impact on the nature of FAs absorbed and ultimately secreted into milk. Pigs and poultry, like humans, have a relatively simple digestive system and absorb FAs in approximately the same proportions as found in their diet. Lipid absorption by cattle and sheep is heavily influenced by rumen microbial activity that hydrogenates (saturates) up to 95% of dietary polyunsaturated fatty acids (PUFAs), making it challenging to increase the PUFA content of ruminant milk or meat. However, increased reliance on fresh herbage in dairy cow diets does elevate the ω-3 content of milk produced [15]–[23], [32].

The U.S. National Organic Program (NOP) requires that lactating cows on certified organic farms receive at least 30% of daily Dry Matter Intake (DMI) from pasture during that portion of the year when pasture grasses and legumes are actively growing, with a minimum of 120 days per year [35]. Pasture and conserved, forage-based feeds account for most of the DMI year-round on a growing portion of organic dairy operations in the U.S. [36].

Although several European studies have compared the composition of organic and conventional milk [18]–[23], [32], there is limited comparative data from the U.S. Also, published U.S. studies are based on relatively limited sampling and reflect milk production during only a portion of a year, hence providing no insight into seasonal changes in milk quality [16], [17]. The two main objectives of the present study were first, to quantify average, annual FA differences between organic and conventional milk in an extensive, 18-month cross-U.S. survey, with attention to regional and seasonal variations, and second, to address the degree to which consumption of predominantly organic dairy products may enhance public health by decreasing dietary ω-6/ω-3 ratios from today’s generally unhealthy levels [5], [7]–[10], [14], [28]–[31], [37]–[48].

## Methods

We selected 14 commercial milk processors from 7 regions throughout the U.S. that produce organic milk products, and usually also conventional milk, pasteurized by either the high-temperature-short-time method (HTST) or by the ultra-high-temperature method (UHT, also known as ultra-pasteurization). The processors were located in the Northwest region (2 HTST, 1 UHT); California (1 HTST, 1 UHT—organic only); Rocky Mountains (1 HTST), Texas (1 HTST); Midwest (1 HTST, 1 UHT—organic only); Mid-Atlantic (1 HTST, 1 UHT—organic only); and Northeast (1 HTST, 2 UHT). These processors receive and market organic milk through the Organic Valley brand, the largest U.S. cooperative of organic farmers, based in La Farge, Wisconsin. Because three of the UHT processors produce only organic milk, we obtained more organic than conventional samples, and more UHT organic samples than UHT conventional samples. From each processor we obtained one fresh, whole-milk sample nominally every month for 18 months, January 2011 through June 2012. A total of 220 organic and 164 conventional samples were taken from either 1-gallon or half-gallon retail containers, transferred to sterile plastic bottles, refrigerated, and shipped with frozen ice packs by overnight courier to Silliker, Inc., an ISO/IEC 17025 accredited lab in Chicago Heights, Illinois. Analyses for FA and total fat used method AOAC 996.06, revised 2001 [49], with capillary column Supelco SP-2560, 100 m×0.25 mm, 0.2 µm film. The lab did not report non-quantifiable amounts defined as < 0.001 g/100 g.

### Statistical Analysis

Digital laboratory data were transferred automatically to an Excel spreadsheet and spot-verified by one of us against printed lab reports. The data are available from authors MAL and DRD. Statistical analyses used NCSS 2004 software (Kaysville, Utah), plus Excel 2003 for some descriptive statistics and *t* distributions. Excel results were verified with NCSS [50]. Primary data were inspected with scatter plots and normal probability plots, and small numbers of clear outliers with large deviations from normality were removed as follows: First, 6 milk samples with total fat ≤ 2.52 and ≥4.17 g/100 g, leaving 218 organic and 160 conventional samples; then 7 extreme outlier values (g/100 g) for individual FA 12:1 (0.046), 18:2 linoleic acid (LA) (0.006), 20:1 (0.026, 0.026, 0.032), and 20:5 (eicosapentaenoic acid, EPA) (0.041, 0.084). The removed outliers deviated from the respective FA means by 5 to 70 SDs, and represent 0.07% of all values examined. Differences between means were evaluated by 2-tailed *t* test for approximately normal distributions or by 2-tailed Mann-Whitney test, as indicated. Mann-Whitney tests were used for non-normal distributions and those with insufficient numbers of samples to evaluate normality.

Ideally our data would have been amenable to ANOVA-based methods, but we chose alternative methods because of complex data that could not be consistently transformed to normality with equal variances, requiring both parametric and non-parametric methods. There are also some 2- and 3-fold differences in the numbers of organic and conventional samples, and in the number of regional samples. Because of multiple comparisons of organic vs. conventional FA concentrations in 2 tables, about 2 findings of *P* = 0.05 and 0.5 finding of *P* = 0.01 can be expected there by chance alone, but our key findings are highly significant (*P*<0.0001) and not vulnerable to Type-I errors (incorrectly rejecting the null hypothesis). To prevent or minimize potentially biased statistical comparisons caused by possible correlation between monthly FA concentrations, we limited statistical comparisons of monthly data between organic and conventional samples to individual months or groups of 6 months. To compare coefficients of variation we used the method by Forkman [51].

We calculated U.S. average fatty acid concentrations and ratios as means of samples from all 12 months in 2011 and all 7 geographical regions, calculated separately for organic and conventional samples. For regional concentrations and ratios, we averaged all 12 monthly samples separately for each region and for both production methods. Similarly, we calculated seasonal concentrations and ratios by averaging samples from all 7 geographical regions, separately for 18 months in 2011–2012 and for both production methods. The numbers of samples are shown in the resulting tables, figures, or figure caption.

Three ratios of FAs in conventional versus organic milk are reported. LA/ALA is the simplest ratio and represents the major ω-6 and ω-3 FAs in milk and human diets. The most commonly encountered ratio of ω-6 and ω-3 FAs is total ω-6/total ω-3, where the totals may include, besides LA and ALA, arachidonic acid (AA, ω-6), EPA (ω-3), DHA (ω-3) and possibly docosapentaenoic acid (DPA) (ω-3) and other minor FAs. Our inclusive ratio ω-6/ω-3 includes primarily LA (ω-6), 8,11,14-eicosatrienoic acid (20:3, ω-6), AA (ω-6), ALA (ω-3), EPA (ω-3), and DPA (ω-3), plus infrequently reported, small amounts of 18:3 γ-linolenic acid (ω-6), 20:2 eicosadienoic acid (ω-6), 22:2 docosadienoic acid (ω-6), 22:4 docosatetraenoic acid (ω-6), 18:4 moroctic acid (ω-3), 20:3 11,14,17-eicosatrienoic acid (ω-3), and DHA (ω-3). To more fully reflect the variations in levels of health-promoting dairy FAs, we include a third ratio in which we add to the ω-3 total the amount of CLA (18:2 conjugated). This FA is widely accepted as beneficial for heart health and prevention of cancer [52]–[54].

### Diet Scenarios and LA/ALA Ratios

To estimate the impact on dietary ω-6/ω-3 ratios of replacing conventional with organic dairy products, we calculated LA/ALA ratios for hypothetical diets of moderately active women, age 19–30, selected in part because of the importance of ω-3 FA during women’s child-bearing years. We limited this estimate to LA and ALA, because of the lack of reliable U.S. Department of Agriculture (USDA) composition data for EPA, DPA, and DHA in non-dairy foods. In addition, LA accounts for 90% of total ω-6 in both organic and conventional milk, and ALA accounts for 79% to 80% of total ω-3. Our estimated impacts on LA/ALA of a switch from conventional to organic dairy foods underestimates the full array of health benefits following such a switch, because CLA and long-chain, ω-3 FAs fall outside the LA/ALA ratio.

Table 1 shows model diets containing hypothetical servings of 4 common dairy foods in 3 diet scenarios—low-fat, average-fat, and high-fat (respectively 20%, 33%, and 45% of energy from fat). Except for the low-fat scenario, the “Moderate dairy intakes” follow the recommended 3 daily servings of milk and milk products in the Dietary Guidelines for Americans [1]. The moderate-dairy, low-fat scenario uses reduced amounts of 3 of the 4 dairy foods, thereby reducing its content of fat and other nutrients from dairy products, e.g., for calcium, from 1040 mg to 850 mg. (However, the reduced dairy intake adds nutrients from 128 kcal of additional non-dairy foods needed to keep total energy at 2,100 kcal.) To quantify the likely maximum benefit possible from consuming milk products with enhanced FA profiles, we use whole milk and full-fat cheese in the diet scenarios, instead of reduced-fat versions. For the recommended 3 cups fluid milk equivalent we use 1.5 cups milk + 1.5 ounces cheese (1 cup fluid milk equivalent) + 6 ounces low-fat yogurt with fruit (about 0.5 cup fluid milk equivalent). Ice cream is categorized separately as “dairy dessert” [1].

**Table 1:** Hypothetical scenarios of dairy fat intake for a moderately active woman, age 19 to 30.

	Total	Milk,	Cheese,	Ice	Yogurt,	Energy	Energy	Energy from
	Energy,	cups	oz.	Cream,	Low fat	from Fat,	From Dairy	Other Fat,
	kcal			0.5 cup	6 oz.	kcal	Fat, kcal*	kcal
**Low-Fat Diet (20% of Energy)**								
Moderate dairy intake	2,100	1.25	1.00	0.75	1.00	420	239	181
High dairy intake	2,100	1.50	1.50	1.00	1.00	420	313	107
**Average-Fat Diet (33% of Energy)**								
Moderate dairy intake	2,100	1.50	1.50	1.00	1.00	693	313	380
High dairy intake	2,100	2.00	3.00	1.00	1.00	693	472	221
**High-Fat Diet (45% of Energy)**								
Moderate dairy intake	2,100	1.50	1.50	1.00	1.00	945	313	632
High dairy intake	2,100	2.00	3.00	2.00	1.00	945	536	409

^*^ Based on the following serving sizes and USDA data:

Milk 1 cup (244 g), 3.25 g fat/100 g.

Cheddar cheese 1 oz. (28.35 g), 33.14 g fat/100 g.

Vanilla ice cream 0.5 cup (66 g), 11.0 g fat/100 g.

Low-fat yogurt with fruit 6 oz. (170.1 g), 1.41 g fat/100 g.

8.79 kcal/g dairy fat.

In Table 1 “Energy from fat”  = 2100 kcal for a moderately active woman [1] × 20%, 33% or 45%; “Energy from dairy fat”  =  serving weight × fat content/100 g×8.79 kcal/g, summed over the 4 foods, using the footnoted values from USDA’s National Nutrient Database for Standard Reference [55]; “Energy from other fat”  =  “Energy from fat” − “Energy from dairy fat.”

We converted the dairy and non-dairy fat-energy values from Table 1 into fat weights using USDA’s 8.79 kcal/g for dairy fat and 8.9 kcal/g for typical non-dairy fat, and then calculated the amounts of LA and ALA contained in these dairy and non-dairy sources. For dairy fat, we used the average amounts of LA, ALA, and total FAs found in this research, plus the conversion from milk FA weight to milk fat weight (0.933 g FA/g fat) [56]. For non-dairy fat, we used USDA data to calculate the amounts of LA and ALA per 100 kcal of fat in 8 foods—McDonald’s French fries, plain tortilla chips, higher-fat chocolate chip cookies, soy oil (salad or cooking), regular stick margarine, ground chicken, composite pork cuts, and ground beef (15% fat).

We selected these 8 foods because they are commonly consumed and USDA reports amounts of their fully differentiated LA (*cis,cis*-18:2 n-6) and ALA (*cis,cis,cis-*18:3 n-3) [55]. On average they contain 23.23 g LA and 1.841 g ALA per 100 kcal fat, which we used to calculate the amount of LA and ALA in “other” (non-dairy) fat in Table 1.

To estimate the effect of reducing LA intakes, we made substitutions for 3 of the above 8 foods—pita chips instead of tortilla chips (LA/ALA  =  20, instead of 40 for tortilla chips), canola oil instead of soy oil, and canola oil margarine instead of regular margarine, all with available fully differentiated data for LA and ALA [55]. These 3 substitutions yield average contents for non-dairy fat of 13.84 g LA (a 40% reduction) and 2.731 g ALA (a 48% increase) per 100 kcal of fat.

### Fatty Acids in Fish Compared to Other Sources

The Dietary Guidelines for Americans [1] recommend consumption of about 8 ounces per week of a variety of fish (about twice the average U.S. consumption), with up to 12 ounces per week for women who are pregnant or breastfeeding. The fish varieties may include those with higher and lower amounts of EPA and DHA, but should include some with higher amounts, to achieve an average intake of 250 mg/day of EPA + DHA. Because fish also contain LA, ALA, and DPA, we used USDA data [55] to estimate the amounts of these 5 FAs in fish and compared them with amounts in the dairy products and non-dairy sources used in our dietary scenarios. We selected 7 representative fish with available data for fully differentiated ALA and (in most cases) LA.

## Results

Table 2 shows FA concentrations in conventional and organic milk, averaged over a full calendar year of milk production (2011). The complete 18-month test period through June 2012 is used to assess seasonal variations. Table 2 includes sums of saturated, monounsaturated, and polyunsaturated FA, as well as ω-3, ω-6, and *trans* FA, plus CLA, CLA + ALA, and various ratios of FA levels, such as LA/ALA and ω-6/ω-3. FA names include the number of carbon atoms followed by a colon and the number of double bonds. Table 2 also shows descriptive statistics, including coefficients of variation (CV  =  SD/mean), a measure of nationwide percentage variation during 2011. Supplemental Table S1 reports the same values expressed as a percentage of total FAs. As expected, percentage variations (CVs) tend to be smaller in Table S1 than in Table 2, especially for the dominant fractions, total saturated FA (∼67%) and monounsaturated FA (∼25%). Supplemental Figures S1 and S2 illustrate key concentrations and ratios.

**Table 2:** Fatty acids in retail whole milk (g/100 g), 12 months ending December 2011.

		Organic					Conventional					Org/Conv	*P*(differ-ence)*
		Mean	n	SD	CV	SE	Mean	n	SD	CV	SE		
	Total triglyceride (calculated)	3.276	143	0.158	4.8%	0.013	3.265	108	0.133	4.1%	0.013	1.00	0.56
	Total fatty acids	3.108	143	0.150	4.8%	0.013	3.098	108	0.127	4.1%	0.012	1.00	0.58
**Saturated fatty acids**													
	4:0 butyric	0.0751	143	0.0062	8.3%	0.0005	0.0738	107	0.0062	8.4%	0.0006	1.02	0.10
	6:0 caproic	0.0607	143	0.0066	11%	0.0006	0.0577	108	0.0055	9.5%	0.0005	1.05	0.00014
	8:0 caprylic	0.0407	143	0.0050	12%	0.0004	0.0384	108	0.0035	9.2%	0.0003	1.06	0.00007
	10:0 capric	0.1288	143	0.0316	25%	0.0026	0.1241	108	0.0283	23%	0.0027	1.04	0.23
	11:0 undecylic	0.0026	85	0.0009	36%	0.0001	0.0026	84	0.0008	32%	0.0001	0.98	0.69
	12:0 lauric	0.1059	143	0.0131	12%	0.0011	0.0990	108	0.0100	10%	0.0010	1.07	0.00001
	14:0 myristic	0.3490	143	0.0251	7.2%	0.0021	0.3274	108	0.0239	7.3%	0.0023	1.07	0.00000
	15:0 pentadecanoic	0.0402	143	0.0036	9.0%	0.0003	0.0355	108	0.0039	11%	0.0004	1.13	0.00000
	16:0 palmitic	0.9344	143	0.0798	8.5%	0.0067	0.8995	108	0.0519	5.8%	0.0050	1.04	0.00010
	17:0 margaric	0.0243	143	0.0022	8.9%	0.0002	0.0216	108	0.0025	12%	0.0002	1.12	0.00000
	18:0 stearic	0.3434	143	0.0390	11%	0.0033	0.3559	108	0.0362	10%	0.0035	0.96	0.010
	20:0 arachidic	0.0064	141	0.0014	21%	0.0001	0.0055	107	0.0010	19%	0.0001	1.17	0.00000
	22:0 behenic	0.0040	105	0.0010	24%	0.0001	0.0032	61	0.0013	41%	0.0002	1.25	0.00001
	24:0 lignoceric	0.0025	73	0.0010	40%	0.0001	0.0024	23	0.0009	39%	0.0002	1.03	0.75
	Total saturated†	2.116	143	0.120	5.7%	0.010	2.043	108	0.095	4.7%	0.009	1.04	0.00000
**Monounsaturated fatty acids**													
	14:1 myristoleic	0.0290	143	0.0038	13%	0.0003	0.0269	108	0.0038	14%	0.0004	1.08	0.00002
	16:1 palmitoleic	0.0468	143	0.0067	14%	0.0006	0.0467	108	0.0069	15%	0.0007	1.00	0.83
	17:1 margaroleic	0.0080	137	0.0014	17%	0.0001	0.0070	106	0.0015	21%	0.0001	1.14	0.00000
	18:1 incl. oleic	0.6505	143	0.0533	8.2%	0.0045	0.7074	108	0.0486	6.9%	0.0047	0.92	0.00000
	20:1 incl. gadoleic	0.0071	126	0.0026	37%	0.0002	0.0067	96	0.0025	38%	0.0003	1.05	0.34
	Total monounsaturated†	0.7410	143	0.0547	7.4%	0.0046	0.7944	108	0.0491	6.2%	0.0047	0.93	0.00000
**ω-3 fatty acids**													
	18:3 α-linolenic, ALA	0.0255	143	0.0040	16%	0.0003	0.0159	108	0.0059	37%	0.0006	1.60	0.00000
	20:5 eicosapentaenoic, EPA	0.0033	104	0.0012	35%	0.0001	0.0025	43	0.0010	41%	0.0002	1.33	0.00009
	22:5 docosapentaenoic, DPA	0.0044	120	0.0012	28%	0.0001	0.0037	70	0.0010	26%	0.0001	1.18	0.00008
	Total ω-3†	0.0321	143	0.0061	19%	0.0005	0.0198	108	0.0084	43%	0.0008	1.62	0.00000
**ω-6 fatty acids**													
	18:2 linoleic, LA	0.0639	143	0.0079	12%	0.0007	0.0856	107	0.0146	17%	0.0014	0.75	0.00000
	20:3 8,11,14-eicosatrienoic (γ)	0.0032	110	0.0010	32%	0.0001	0.0043	92	0.0012	27%	0.0001	0.75	0.00000
	20:4 arachidonic, AA	0.0048	118	0.0014	29%	0.0001	0.0058	91	0.0016	28%	0.0002	0.83	0.00001
	Total ω-6†	0.0711	143	0.0093	13%	0.0008	0.0948	107	0.0166	18%	0.0016	0.75	0.00000
	Total Polyunsaturated†	0.1037	143	0.0126	12%	0.0011	0.1147	107	0.0165	14%	0.0016	0.90	0.00000
***trans*** ** fatty acids**													
	*trans*-16:1 *trans*-palmitoleic	0.0131	143	0.0023	17%	0.0002	0.0117	108	0.0019	17%	0.0002	1.12	0.00000
	*trans*-18:1 incl. elaidic	0.0846	143	0.0235	28%	0.0020	0.0906	108	0.0143	16%	0.0014	0.93	0.00022‡
	*trans*-18:2 octadecadienoic	0.0257	143	0.0084	33%	0.0007	0.0243	107	0.0061	25%	0.0006	1.05	0.17
	Total *trans* §	0.1254	143	0.0292	23%	0.0024	0.1281	108	0.0190	15%	0.0018	0.98	0.40
**Conjugated linoleic acid, CLA**													
	18:2 conjugated	0.0227	143	0.0084	37%	0.0007	0.0192	106	0.0049	25%	0.0005	1.18	0.00019
**Sum**													
	ALA + CLA	0.0481	143	0.0105	22%	0.0009	0.0347	108	0.0098	28%	0.0009	1.39	0.00000
**Ratios**													
	LA/ALA	2.568	143	0.544	21%	0.046	6.272	107	2.485	40%	0.240	0.41	0.00000
	ω-6/ω-3	2.276	143	0.469	21%	0.039	5.774	107	2.520	44%	0.244	0.39	0.00000
	ω-3/ω-6	0.456	143	0.083	18%	0.007	0.219	107	0.124	57%	0.012	2.08	0.00000
	ω-6/(ω-3 + CLA)	1.353	143	0.345	26%	0.029	2.742	107	1.223	45%	0.118	0.49	0.00000

^*^ Calculated by *t* test except as noted. Because of multiple comparisons, about 2 findings of *P* = 0.05 and 0.5 finding of *P* = 0.01 can be expected by chance.

^†^ These group means (means of sums of saturated, monounsaturated, or polyunsaturated FA) are biased slightly low, because they include some sums containing unreported small values (< 0.001) treated as zero. In contrast, when n is less than the number of samples (organic n<143, conventional n<108), means of individual FA are biased slightly high by omission of unreported small values. Thus these group means are slightly less than the sum of means of the individual FA.

^‡^ Calculated by Mann-Whitney test due to non-normal distributions with medians 0.080 (organic) and 0.091 (conventional). (*P* = 0.020 calculated by *t* test).

^§^ The *trans* FA group mean exceeds the sum of individual *trans* FA, because it includes small amounts of *trans*-14:1 omitted from the table due to small numbers of reported values (42 organic, 27 conventional).

There were small or negligible differences between organic and conventional milk in total FAs (< 1%, not statistically significant), and in total saturated (4% higher in organic), monounsaturated (7% lower in organic), and *trans* FAs (2% lower in organic, not statistically significant). However, 12-month-average concentrations of total PUFAs, total ω-6, and LA were significantly higher in conventional compared to organic milk (by 11%, 33%, and 34% respectively, all *P*<0.0001), while concentrations of total ω-3, ALA and CLA were significantly higher in organic compared to conventional milk (by 62%, 60%, and 18% respectively, *P*<0.0001, <0.0001, and < 0.001).

Similarly, 12-month average concentrations of the more active, longer-chain ω-3 FAs EPA and DPA were significantly higher in organic milk compared to conventional milk (33% and 18% respectively, both *P*<0.001). Data for DHA are not shown, because over the 18 months of testing, quantifiable levels (≥ 0.001 g/100 g) were found in only 4 of 218 organic and 2 of 160 conventional samples. Our results are similar to other studies that report significant levels of EPA and DPA in dairy products, but little or no DHA [17], [18], [21]. Circulating EPA and DHA in cow blood are believed to have extremely low uptake in the udder. It is speculated that EPA, but very little DHA, is synthesized de novo in the udder, and that this synthesis is the primary source of EPA in cow milk [57].

As a result of the observed composition differences, the 2011 average ratios LA/ALA, ω-6/ω-3, and ω-6/(ω-3 + CLA) are much higher in conventional compared to organic milk—2.4-fold higher for LA/ALA, 2.5-fold higher for ω-6/ω-3, and 2.0-fold higher for ω-6/(ω-3 + CLA) (Table 2, all *P*<0.0001).

### Regional Differences

**Figure 1:**
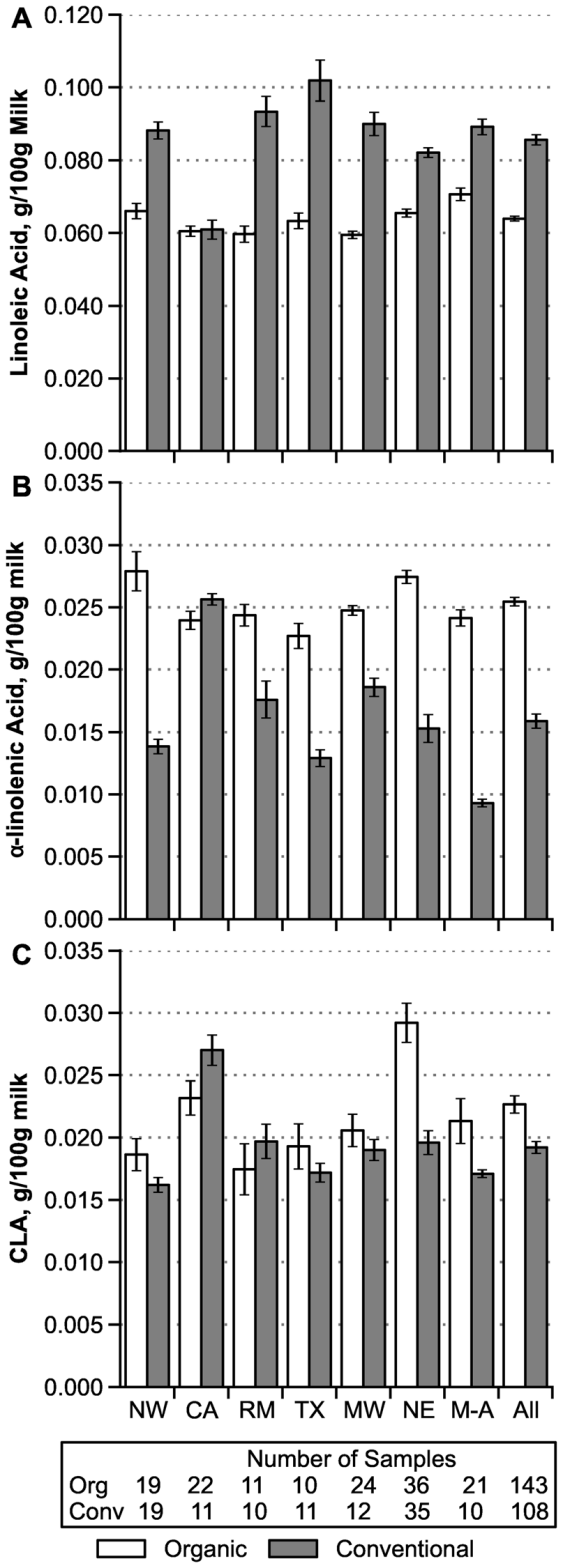
Regional variation in fatty acid content of retail whole milk, g/100 g (12-month average ± SE). **A:** Linoleic acid (LA, ω-6). **B:** α-linolenic acid (ALA, ω-3). **C:** Conjugated linoleic acid. Abbreviations: NW  =  Northwest, CA  =  California, RM  =  Rocky Mountain, TX  =  Texas, MW  =  Midwest, NE  =  Northeast, M-A  =  mid-Atlantic. Numbers of samples apply to panels B and C; for panel A conventional NE is 34 and All is 107. For LA and ALA, all differences between organic and conventional contents are statistically significant by Mann-Whitney test (*P*<0.005) except for the CA region (*P*≥0.10). For CLA no such differences are statistically significant (*P*>0.08) except for the NE region and All regions (*P*<0.001).

**Figure 2:**
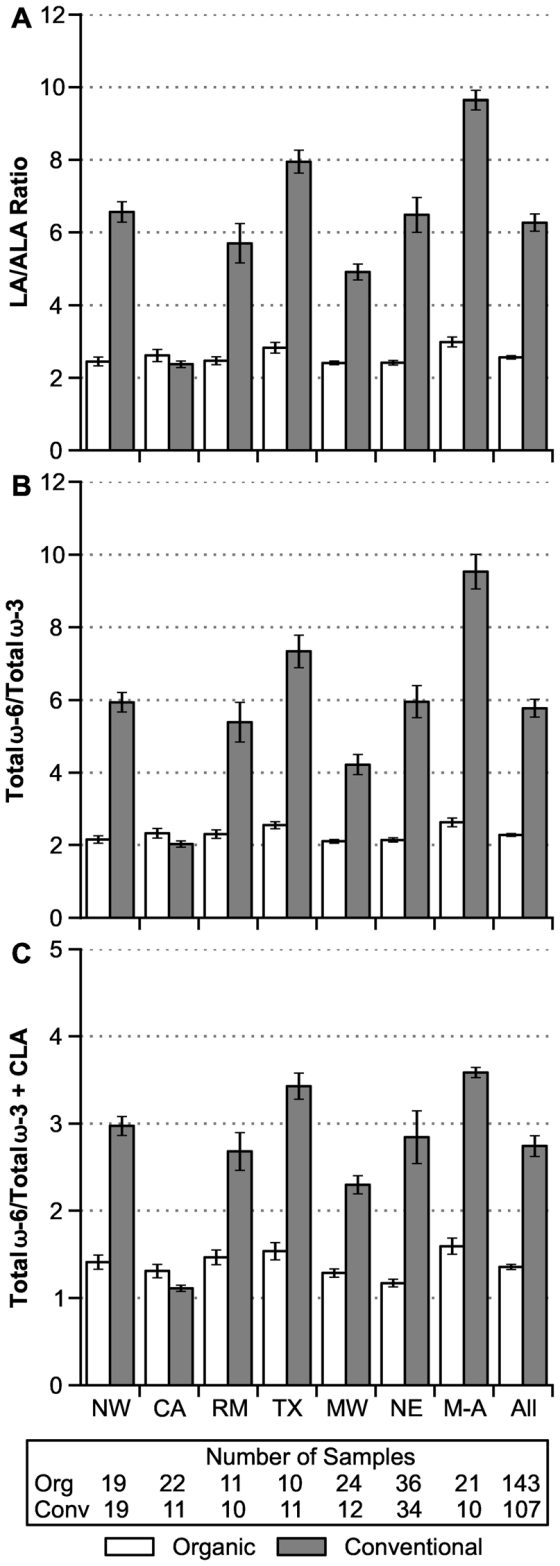
Regional variation in ratios involving ω-6 and ω-3 fatty acids (12-month average ± SE). **A:** Linoleic acid/α-linolenic acid (LA/ALA). **B:** Total ω-6/total ω-3. **C:** Total ω-6/(total ω-3 + CLA). Abbreviations: Same as Fig. 1. Numbers of samples apply to all panels.

National 12-month averages show a few clear differences between regions in both the concentrations of LA, ALA, and CLA (Figure 1) and in the FA ratios LA/ALA, ω-6/ω-3, and ω-6/(ω-3 + CLA) (Figure 2). Most notably, conventional milk from California (CA) (represented in this study by its far northern Humboldt County) is unusually low in LA and high in ALA and CLA, and it has a FA profile similar to nationwide organic milk. Conversely, conventional milk from the Mid-Atlantic region has unusually high ratios of LA/ALA and ω-6/ω-3. One-way analyses of variance among regions for conventional milk do not consistently conform to assumptions of normality and equal variance, but they suggest that values for the CA region differ from all other regions with high reliability (*P*<0.000001, ANOVA of log-transformed data, followed by Tukey-Kramer pair-wise comparisons). For other comparisons of possible interest in Figures 1 and 2, two values are likely reliably different (*P*<0.05) if their SE error bars are well separated in vertical position.

For some measures in Figures 1 and 2, variability among regions is notably larger for conventional compared to organic samples, as measured by CVs of the regional means. Even excluding the most variable values for conventional milk from CA, CVs are about 3-fold larger in conventional compared to organic samples for ALA (23% vs. 7.6%, *P* = 0.02), LA/ALA (25% vs. 8.8%, *P* = 0.03), and ω-6/ω-3 (29% vs. 8.9%, *P* = 0.015). For CLA, there is an opposite trend toward greater regional variability in organic milk (CV  = 18% vs. 8.2% without CA, *P* = 0.08). There is negligible difference in regional variability for LA (organic 6.5% vs. conventional 7.2% without CA, *P* = 0.8).

### Seasonal Differences

**Figure 3:**
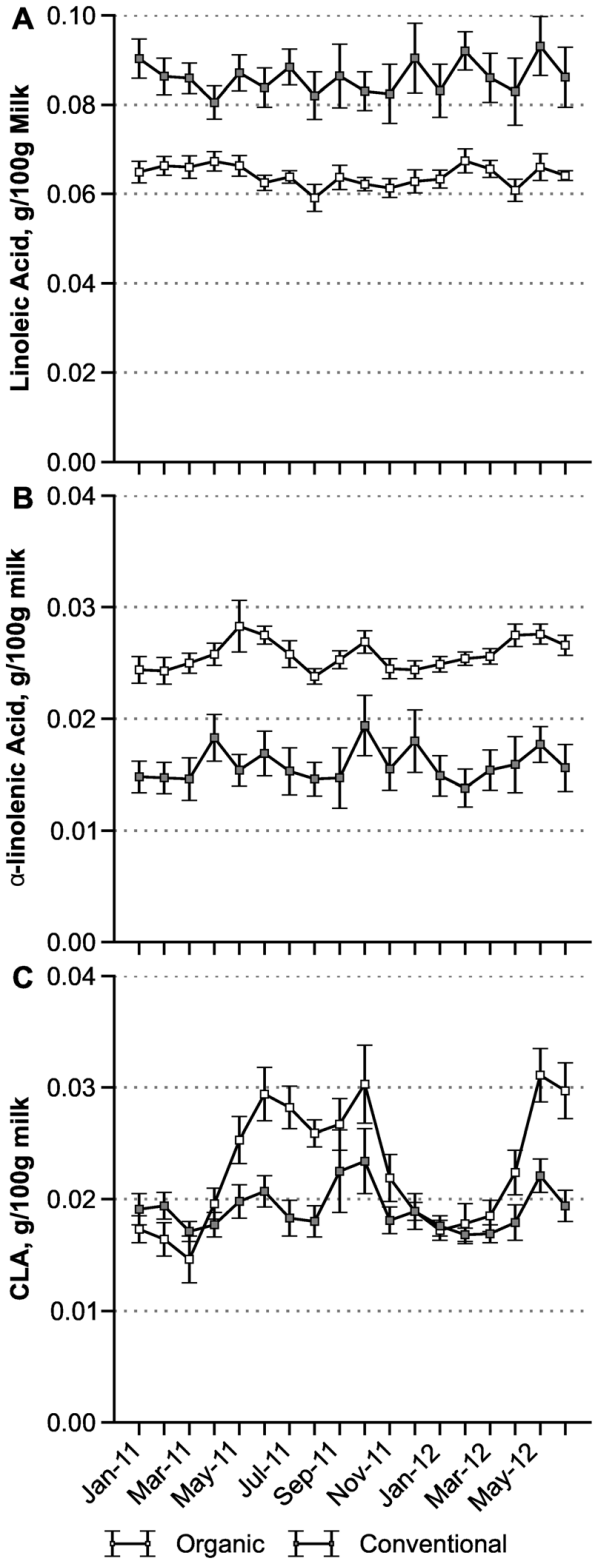
Seasonal variation in polyunsaturated fatty acid content of retail whole milk, g/100 g, 18 monthly averages ± SE. A: Linoleic acid (LA, ω-6). B: α-linolenic acid (ALA, ω-3). C: Conjugated linoleic acid (CLA). The number of monthly samples in all panels is 10 to 13 for organic and 7 to 10 for conventional milk, except 5 conventional in October 2011. For LA and ALA, all differences between organic and conventional contents are statistically significant by Mann-Whitney test (*P*<0.02).

Figure 3 shows monthly variations in the national average composition of organic and conventional milk over the full 18 months of the study. Concentrations of LA and ALA, and the differences between them, are similar across time (Figures 3A and 3B). In contrast, there is substantial seasonal variation in CLA, especially in organic milk (Figure 3C). We compared the national average CLA concentrations during the “summer” calendar months of May–October (May–Oct. 2011 and May–June 2012) with those during the “winter” months of November–April (Jan.–Apr. and Nov.–Dec. 2011, plus Jan.–Apr. 2012). In organic milk the CLA concentration was 55% higher in summer than in winter, with averages ± SE (in mg/100 g) of 0.0283±0.0008 in summer vs. 0.0183±0.0005 in winter (*P* = 0.000000 by Mann-Whitney test, n = 96 and 122 respectively). In conventional milk the summer increase was a much smaller 12% (0.0200±0.0006 vs. 0.0179±0.0003 mg/100, *P* = 0.0074, n = 67 and 90).

We also compared the above national average CLA concentrations between organic and conventional samples, separately by season. In the summer months the organic average was 42% higher (0.0283±0.0008 vs. 0.0200±0.0006 mg/100 g, *P* = 0.000000 by Mann-Whitney test, n = 96 and 67 respectively). But in the winter months the difference between organic and conventional samples was negligible (respectively 0.0183±0.0005 vs. 0.0179±0.0003 mg/100 g, *P* = 0.86, n = 122 and 90). Averaged over the full year of 2011, the CLA concentration was 18% higher in organic compared to conventional samples (Table 2).

Similarly for ALA in organic milk, Figure 3 shows a small (4%) but statistically significant increase in summer compared to winter (0.0262 ± SE 0.0003 vs. 0.0252 ± SE 0.0003 mg/100 g, *P* = 0.022, by Mann-Whitney test, n = 95 and 122). A corresponding 2.6% summer increase in conventional milk is not statistically significant (*P* = 0.54, n = 69 and 96). Differences between organic and conventional milk are highly reliable in both summer and winter (*P* = 0.000000) and reliable for each of the 18 months (*P* ≤ 0.020).

For LA, we find no evidence for seasonal variability in national averages for either organic or conventional milk (*P* = 0.13 and 0.85, respectively, by Mann-Whitney tests). However, LA differences between organic and conventional milk are highly reliable in both summer and winter (*P* = 0.000000) and reliable for each of the 18 months (*P* ≤ 0.017).

In Figure 3 the smaller variability of 18 monthly means in organic compared to conventional milk is statistically significant for ALA (CV = 5.2% vs. 9.8%, *P* = 0.014), but not for LA (CV = 3.7% vs. 4.2%, *P* = 0.58). For CLA monthly variability is larger in organic samples, 24% vs. 10%, *P* = 0.0013.

Diet Scenarios and LA/ALA

We analyzed a series of diet scenarios to assess the impact of conventional and organic dairy products on total dietary LA/ALA ratios (see Table 1). The top half of Table 3 shows the resulting intakes of LA and ALA in hypothetical diets for an adult woman consuming an average portion of energy from fat (33%) and typical non-dairy fat sources. The highest LA/ALA ratio of 11.3 occurred in the diet with moderate amounts (3 servings per day, fluid milk equivalent) of conventional whole milk and other mostly full-fat dairy products. Compared to this baseline value, the ratios decreased to 10.0 with either a switch to organic dairy products or a 50% increased consumption of conventional dairy products (4.5 servings per day, fluid milk equivalent). A combination of both changes (high intake of organic dairy products) reduced the LA/ALA ratio to 7.8. Expressed as a percentage of the reduction needed to reach a goal of LA/ALA  =  2.3, either change alone (switch to organic or increase in conventional dairy consumption) achieves about 15% of the needed reduction, and a dual switch to organic and increased consumption of organic dairy products accomplishes about 40% of the necessary reduction.

**Table 3:** LA and ALA contents of hypothetical average-fat diets with typical and low-LA non-dairy fat sources.

	LA from	ALA from	LA from	ALA from	Total	Total	Total LA/	Decrease	Decrease from Base-
	Dairy Fat,	Dairy Fat,	Other	Other	LA,	ALA,	Total ALA	from Base-	line as % of Decrease
	g*	G*	Fat, g†	Fat, g†	g	g		line LA/ALA	Needed to Reach
									LA/ALA = 2.3
**With Typical Non-Dairy Fat Sources**									
**Conventional Dairy**									
Moderate intake	0.92	0.17	9.91	0.79	10.83	0.96	11.33	--	--
High intake	1.38	0.26	5.77	0.46	7.15	0.71	10.01	1.31	15%
**Organic Dairy**									
Moderate intake	0.68	0.27	9.91	0.79	10.59	1.06	10.01	1.32	15%
High intake	1.03	0.41	5.77	0.46	6.80	0.87	7.83	3.50	39%
**With Low-LA Non-Dairy Fat Sources**									
**Conventional Dairy**									
Moderate intake	0.92	0.17	5.90	1.17	6.82	1.34	5.11	6.22	69%
High intake	1.38	0.26	3.44	0.68	4.82	0.94	5.15	6.17	68%
**Organic Dairy**									
Moderate intake	0.68	0.27	5.90	1.17	6.59	1.44	4.58	6.74	75%
High intake	1.03	0.41	3.44	0.68	4.47	1.09	4.10	7.23	80%

^*^ Based on LA, ALA, and total FA from Table 2, 8.79 kcal/g dairy fat, and 0.933 g milk FA/g milk fat.

E.g., LA 0.92 = 313 (Table 1) × 0.0856×0.933/8.79/3.098.

†Based on 23.23 g LA and 1.841 g ALA per 100 kcal non-dairy fat, 8.9 kcal/g non-dairy fat.

E.g., LA 9.91 = 380 (Table 1) × 23.23/8.9/100.

Corresponding calculations for low-LA non-dairy fat use 13.84 g LA and 2.731 g ALA per 100 kcal non-dairy fat.

The bottom half of Table 3 shows the effects of the same variations in types and amounts of milk in the context of a diet with reduced amounts of LA from non-dairy sources. In these low-LA scenarios, we substituted pita chips for tortilla chips, canola oil for soy oil, and canola-oil margarine for regular margarine, with no change in French fries, chocolate chip cookies, chicken, pork, or beef. These reductions in LA intake consistently reduced the overall dietary LA/ALA ratios to about 4 or 5, or 68% to 80% of the way toward 2.3, even with moderate consumption of conventional, mostly full-fat dairy products.

**Figure 4:**
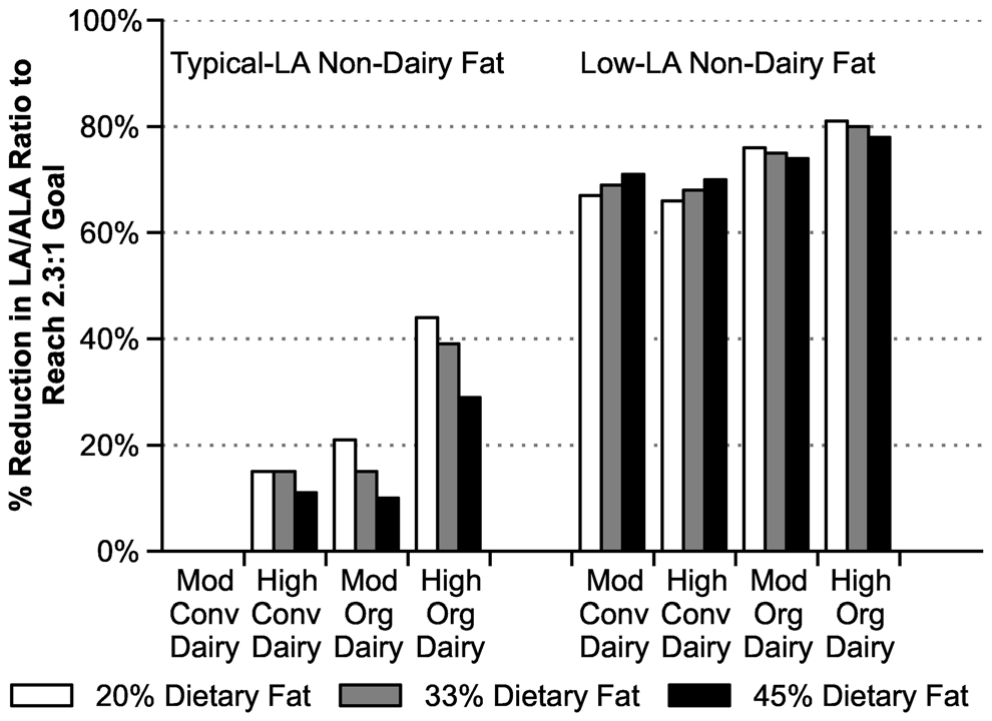
Percent progress toward a dietary LA/ALA ratio of 2.3 for hypothetical diets of an adult woman, relative to diets containing moderate amounts of conventional dairy products. The diets contain non-dairy fat sources with typical (left side) and low (right side) amounts of LA in the context of total dietary fat contributing 20%, 33%, or 45% of energy. Abbreviations: Mod  =  moderate, Conv  =  conventional, Org  =  organic.

Figure 4 shows the above percentages of progress toward an LA/ALA ratio of 2.3, along with the results of additional scenarios in which we varied the baseline intake of total fat (to 20% and 45% of energy, in addition to 33%.

### Fatty Acids in Fish Compared to Other Sources

Table 4 shows daily average FA contents and LA/ALA ratios of, (a) 8 ounces per week of 7 representative fish varieties (sorted by fat content) and their average, (b) conventional and organic dairy products, and (c) the two non-dairy fat sources used in our dietary scenarios. The serving amounts are based on the Dietary Guidelines for Americans, as previously described. The 7 fish range from poor to excellent sources of EPA and DHA. On average they contain 90 + 155 = 245 mg of EPA + DHA, close to the Dietary Guidelines goal of 250 mg/day from fish [1].

**Table 4:** Daily average contents of 8 oz. per week of cooked fish, with comparison to other daily fat sources.

Food	USDA	Fat	Fat Energy	LA	ALA	EPA	DPA	DHA	LA/ALA
	no.	g	kcal*	g	g	g	g	g	
Tuna, light, canned in water	15121	0.311	2.8	0.005	0.001	0.009	0.001	0.064	7.0
Tilapia, farmed	15262	0.859	7.8	0.092	0.015	0.002	0.019	0.042	6.3
Halibut with skin, Alaska	35188	0.885	8.0	0.006	0.003	0.079	0.017	0.118	2.0
Salmon, sockeye, Pacific	15273	1.508	13.6	0.035	0.018	0.111	0.029	0.211	1.9
Catfish, channel, farmed†	15234	1.926	17.4	0.294	0.021	0.007	0.006	0.022	14.0
Trout, rainbow, farmed†	15240	2.004	18.1	0.181	0.023	0.084	0.035	0.201	7.9
Salmon, Atlantic, farmed†	15236	4.352	39.3	0.350	0.058	0.335	0.153	0.430	6.1
**Average of high- and low-fat fish**		**1.692**	**15.3**	**0.138**	**0.020**	**0.090**	**0.037**	**0.155**	**6.5**
Milk products, conventional‡	--	--	313	0.92	0.170	0.027	0.040	--	5.4
Milk products, organic‡	--	--	313	0.68	0.273	0.035	0.047	--	2.5
Diet scenario non-dairy fat, typical LA§	--	--	380	9.91	0.79	--	--	--	12.6
Diet scenario non-dairy fat, low LA§	--	--	380	5.90	1.17	--	--	--	5.1

^*^ Based on 9.02 kcal/g of fish fat (USDA).

†Cooked fish values, estimated from USDA's raw fish values × 1.2.

‡Fat energy from Table 1 (moderate dairy intake, average- or high-fat diet, 33% or 45% fat energy). FA amounts

calculated from Table 2 values (sample calculation in Table 3).

§Fat energy from Table 1 (moderate dairy intake, average-fat diet, 33% fat energy). FA amounts calculated as

shown in Table 3 sample calculation and footnote.

## Discussion

This study is the first large-scale, national milk-fat composition survey in the U.S. comparing milk from organic and conventional farms. The results are based on 378 samples of organic and conventional milk from 7 regions collected over 18 months.

The sampling protocol allows assessment of the impacts of organic and conventional production systems on milk FA composition, as well as regional and seasonal differences.

A key finding is that the nationwide average ratios of LA/ALA and ω-6/ω-3 in 2011 were 2.6 and 2.3 respectively for organic milk samples, compared to 6.3 and 5.8 for the samples from cows on conventionally managed dairies (*P*<0001). Although the ω-6/ω-3 ratio of both conventional and organic dairy fat is healthier than the ratio of most other commonly consumed fat sources, full-fat organic dairy products offer clear advantages for individuals striving to reduce their overall dietary ω-6/ω-3 ratio.

This study confirms earlier findings that milk from cows consuming significant amounts of grass and legume-based forages contains less LA and other ω-6 FAs and higher concentrations of ALA, CLA, and the long-chain ω-3s EPA and DPA, compared to cows lacking routine access to pasture and fed substantial quantities of grains [17]–[23], [32], [58]. In most countries, lactating cows on organically managed farms receive a significant portion of daily DMI from pasture and conserved, forage-based feeds [18], [19], [21], [23], while cows on conventional farms receive much less. In the most recent U.S. government dairy sector survey, only 22% of cows had access to pasture [59], and for most of these, access was very limited in terms of average daily DMI.

The greater regional variation in conventional compared to organic milk (Figs. 1 and 2) likely arises from large regional variations in the feed sources in lactating cow rations. For example, conventional dairy farms near vegetable oil, soy biodiesel, or ethanol plants are likely to feed byproducts from these plants [60]. Other farms might rely on brewers dried grain (from malting barley) or a wide range of food processing wastes. Organic dairy operations, in contrast, are much more dependent on relatively uniform pasture and forage-based feeds, in part because of the grazing requirement in the NOP rule [35]. Also, certified organic sources of most processing wastes and byproduct feeds are not available in substantial quantities.

The FA similarities between conventional and organic milk from our CA region (Figs. 1 and 2) were unexpected. These conventional and organic milk samples came from the Humboldt County area in far Northern CA, a coastal region where both types of dairy farms graze cattle for over 250 days per year. This heavy reliance on pasture contrasts sharply to the near-zero access to pasture on most conventional dairy farms throughout CA’s central valley (the major dairy production region in CA) [61].

In the U.K. and much of Europe, cows on conventional farms have routine access to grazing, although less so than cows on organic farms in the U.S. In a study of organic and conventional dairy farms in North England, grazing accounted for 37% of average DMI on 29 organic farms, compared to 20% on conventional outdoor farms and 3% on conventional indoor operations (annual averages) [19]. During the cold season, indoor-period organic dairy diets had a higher ratio of conserved forage to concentrate compared to conventional dairy diets [18], [22].

On most U.S. organic dairy farms, pasture and on-farm, forage-based feeds account for more than 30% to well over one-half of daily DMI for much of the year [36]. In contrast, the proportion of fresh forage in conventional dairy diets has decreased continuously over the last 40 years in the U.S. Grain-based “total mixed rations” now dominate the conventional U.S. dairy sector. In 2007, 94% of dairy farms milking 500 or more cows fed a total mixed ration, as did 71% of high-production dairies (herd average > 20,000 pounds annual milk production per cow) [59]. The differences in feeding regimes between organic and conventional dairy farms (and associated impacts on milk composition) would therefore be expected to be greater in the U.S. than in Europe.

We find little seasonal variability in milk concentrations of LA, ALA, and CLA, with the notable exception of CLA in organic milk (Fig. 3). In organic milk, CLA peaked during May through October and fell back in December through March to levels similar to conventional milk. Other studies show that CLA levels are especially dependent on pasture feeding with immature, nutrient-rich grasses and legumes, and that any form of mechanical harvest and storage leads to some loss of forage quality, affecting especially the CLA content of milk [15], [19]. These findings likely explain why CLA levels in milk fall on most organic farms over the winter, and peak in the spring and summer, when pasture quality is optimized, and why there is little or no seasonal CLA variation in milk from conventional cows with little access to pasture.

Our results and others confirm that there are significant opportunities to improve the FA profile of milk and dairy products. The potential human health benefits stemming from such improvements are less clear and must be evaluated in the context of overall dietary FA intakes and trends.

During the last century in the U.S. and other developed countries, increasing intakes of LA from vegetable oils, especially soy oil used by food processors, account for most of the shift in typical ω-6/ω-3 ratios from a relatively healthy ∼5 in the early 1900s to ∼15 in much of Europe [62] and around 10 to 15 in the U.S. [13], [24], [26], [63]. LA and ALA are the main ω-6 and ω-3 PUFAs and account for respectively 84%–89% and 9%–11% of the total PUFA intake in Western diets [26]. In contrast, intakes of the more potent, longer-chain ω-3 FAs such as EPA, DPA, and DHA are relatively low in most Western diets, caused by low fatty fish intakes.

Many studies and reviews have concluded that reducing dietary ω-6/ω-3 ratios during adulthood will lower risks of CVD [14], [29]–[31], metabolic syndrome and diabetes [8], [38], [41], [43], [44], [64], overweight [7], [28], [37], [39], [48], [62], and violent behavior [40], [41], [65]. One study reported that a group of adults with the highest plasma ALA levels had ∼30% lower incidence of diabetes [44], and a systematic review of lipid-lowering agents concluded that ω-3 FAs are as effective as statin drugs in lowering CVD risk [47].

Expected benefits from reduced dietary ω-6/ω-3 ratios, coupled with increased long-chain ω-3 intakes, are almost certainly greatest for women hoping to bear a child, for pregnant women and their babies, and for infants and children through adolescence [45]–[48]. High ω-6/ω-3 ratios and/or low long-chain ω-3 intakes predispose the developing fetus to a wide range of adverse neurological and immune system disorders, and can also impair the visual system [66]. Recent research shows that high LA/ALA dietary ratios depress long-chain ω-3 levels in the blood of pregnant women by two mechanisms—by depressing the conversion of ALA to long-chain ω-3s and by blocking incorporation of pre-formed, long-chain ω-3s into phospholipids [67]–[71].

Adults are able to convert a small fraction of ALA to EPA, DPA, and—mainly in women—to DHA [67], [68], [70], [72]–[75]. However, excess dietary LA competes with ALA for the enzymes involved in these conversions [70], [71]. One study found that an LA intake of 30 g/day reduces ALA conversion to DHA by ∼40% [71], while others have shown that certain diets allow pre-menopausal women to convert up to 3-fold more DPA to DHA than males [68]. Accordingly, the improved LA/ALA ratio in organic milk (2.6 organic vs. 6.3 conventional) secondarily benefits consumers by enhancing conversion of ALA to long-chain ω-3s.

### Fatty Acids in Fish Compared to Other Sources

Table 4 shows that 8 ounces per week of a variety of fish (represented by the 7-fish average) contains small amounts of LA compared to dairy products, and only 12% and 7% as much ALA as conventional and organic dairy products, respectively. Compared to the more dominant non-dairy sources of PUFAs, fish contributes negligible LA and ALA, and its average LA/ALA ratio of 6.5 is not distinctive. An important implication is that recommended servings of fish cannot significantly alter U.S. dietary ratios of LA/ALA. Fish also cannot greatly alter ω-6/ω-3 ratios that are typically dominated by LA and ALA. However, our dietary scenarios show how LA/ALA ratios and presumably ω-6/ω-3 ratios can be improved by changing the types and amounts of dairy fat, and especially by reducing LA intake.

The most distinctive FA of fish in Table 4 is DHA, which does not occur in plant foods or significantly in cow’s milk. Fish is less unique for EPA and not unique for DPA. Recommended amounts of dairy products, if mostly full-fat, contain about one-third as much EPA as a mixture of fish varieties, and contain as much DPA—or if organic, somewhat more DPA.

### CLA and Other *Trans* Fatty Acids

Although industrially-produced *trans* FAs are recognized as generally harmful, CLA and the other major *trans* FA in cow’s milk are probably beneficial or harmless to humans [76]. The dominant CLA in milk (75%–90%) [16] is *cis*-9,*trans*-11 18:2, known as rumenic acid and shown as “18:2 conjugated” in Table 2. Because CLAs have probable benefits in humans [52], [53] and proven benefits in animals [16], [54], the U.S. Food and Drug Administration does not count them as *trans* FA for food labeling purposes [77]. Conventional dairy products account for about 75% of U.S. CLA consumption [16], and organic production, especially spring pasture, is known to increase CLA levels [18]–[23], [32]. We find an annual average 18% increase.

The major *trans* FA in dairy fat is *trans*-18:1 (included in “*trans*-18:1 incl. elaidic” in Table 2), of which the dominant isomer (25%–75%) is *trans*-11 18:1, vaccenic acid [16]. At the high range of human intakes, vaccenic acid has little or no effect on CVD risk factors [78]. Humans convert about 20% of it to the rumenic acid form of CLA [16]. In our samples, *trans*-18:1 is reduced by 7% in organic milk.

In human plasma, *trans*-16:1 comes almost exclusively from dairy fat and ruminant meats and thus serves as a marker for consumption of these foods. A recent study found that plasma levels are strongly associated with dairy fat consumption and also with broad health benefits—reduced incidence of new-onset diabetes, favorable CVD risk profile, reduced insulin resistance and inflammation (C-reactive protein), and slightly lower body fat [3]. We find that *trans*-16:1 is 12% higher in organic compared to conventional milk.

### Organic Dairy Products and LA/ALA Ratios

In this study, organic production reduced the 12-month-average ω-6/ω-3 ratio of whole milk from the conventional milk level of 5.77 to only 2.28. The 18% higher level of CLA in organic milk is an additional health benefit [52]–[54], as are the generally higher levels of antioxidants in organic milk [18], [19], [22], [23]. But a key question remains—is the shift in FA profiles in organic milk and dairy products sufficient to improve health outcomes? And if so, by how much, and for whom?

Our dietary scenarios show that organic dairy products can improve dietary LA/ALA and ω-6/ω-3 ratios in adults. Because both conventional and organic dairy fat have ω-6/ω-3 ratios superior to most other fat sources in typical Western diets, replacing non-dairy fat with full-fat dairy products, whether conventional or organic, will improve total dietary LA/ALA ratios. Without other changes, increasing dairy fat intake would increase overall dietary fat and calories, an unwelcome outcome for most people. But if coupled with reduced intakes of food products containing vegetable oils and/or other sources of saturated fat, overall fat content and energy intake can remain unchanged or even decline, while dramatically improving the diet’s ALA content and ω-6/ω-3 ratio.

The impact—and importance—of selecting low-LA alternatives to high-LA foods is unmistakable in our dietary scenarios (Table 3 and Figure 4). They focus on women of childbearing age because of the heightened importance of adequate ω-3 intakes during pregnancy and lactation, as well as the need for efficient conversion of ALA to long-chain ω-3s. The scenarios suggest that the LA/ALA ratio can be reduced by ∼30% to 45% of the way toward the target of 2.3 through high consumption of mostly full-fat organic dairy products, compared to moderate (Dietary Guidelines) consumption of corresponding conventional dairy products. But when coupled with partial reduction of high-LA foods, women can achieve ∼80% of the reduction needed to reach a target ratio of LA/ALA ∼2.3.

Our scenarios may be summarized as follows: For adult women consuming typical-LA non-dairy fat sources, an increase from moderate to 50% higher intakes of conventional dairy products alone reduces dietary LA/ALA ratios by about 10 to 15% of the way toward a target ratio of 2.3. Alternatively, a switch to only moderate amounts (3 servings per day) of mostly full-fat, organic dairy products achieves similar reductions. The switch to 50% higher amounts of organic dairy products adds a further roughly 25% increment toward the 2.3 goal (for a total increment near 40%). The additional step of partially choosing low-LA sources of non-dairy fats brings the overall reduction to ∼75–80% of the way toward the 2.3 goal.

We conclude that increasing reliance on pasture and forage-based feeds on dairy farms has considerable potential to improve the FA profile of milk and dairy products. Although both conventional and organic dairies can benefit from grazing and forage-based feeds, it is far more common—and indeed mandatory on certified organic farms in the U.S.—for pasture and forage-based feeds to account for a significant share of a cow’s daily DMI. Moreover, improvements in the nutritional quality of milk and dairy products should improve long-term health status and outcomes, especially for pregnant women, infants, children, and those with elevated CVD risk. The expected benefits are greatest for those who simultaneously avoid foods with relatively high levels of LA, increase intakes of fat-containing dairy products, and switch to predominantly organic dairy products.

## Supporting Information

Figure S1 **Fatty acid content of retail whole milk, g/100 g (12-month average ± SE).** Some SE are too small to be visible. Abbreviations: Sat  =  saturated, Mono  =  monounsaturated, Poly  =  polyunsaturated, LA  =  linoleic acid, ALA  =  α-linolenic acid, CLA  =  conjugated linoleic acid, EPA  =  eicosapentaenoic acid, DPA  =  docosapentaenoic acid. Differences between organic and conventional contents are statistically significant by Mann-Whitney test (*P*<0.001) except for Total and *Trans* fatty acids (*P* > 0.40).
(TIF)

Figure S2 **Ratios involving ω-6 and ω-3 fatty acids (12-month average ± SE).** Low ratios denote increased amounts of ω-3 and other fatty acids that are commonly low in modern diets and are beneficial to heart, brain, eye, and other tissues and functions. Abbreviations: LA  =  linoleic acid, ALA  =  α-linolenic acid, CLA  =  conjugated linoleic acid. Differences between organic and conventional ratios are statistically significant (*P*<0.0001).
(TIF)

Table S1 (DOC)

## References

[1] U.S. Department of Agriculture (2010) Dietary guidelines for Americans, 7^th^ Edition. Available: http://www.cnpp.usda.gov/DGAs2010-PolicyDocument.htm. Accessed 2013 Mar 15.

[2] Chardigny J-M, Destaillats F, Malpuech-Brugère C, Moulin J, Bauman DE, et al (2008) Do *trans* fatty acids from industrially produced sources and from natural sources have the same effect on cardiovascular diseases risk factors in healthy subjects? Results of the *trans* Fatty Acids Collaboration (TRANSFACT) study. Am J Clin Nutr 87: 558–566. 18326592 10.1093/ajcn/87.3.558

[3] Mozaffarian D, Cao H, King IB, Lemaitre RN, Song X, et al (2010) *Trans*-palmitoleic acid, metabolic risk factors, and new-onset diabetes in U.S. adults. Ann Intern Med 153: 790–799. 21173413 10.1059/0003-4819-153-12-201012210-00005 PMC3056495

[4] German JB, Gibson RA, Krauss RM, Nestel P, Lamarche B, et al (2009) A reappraisal of the impact of dairy foods and milk. Eur J Nutr 48: 191–203. 19259609 10.1007/s00394-009-0002-5 PMC2695872

[5] Elwood PC, Pickering JE, Givens DI, Gallacher JE (2010) The consumption of milk and dairy foods and the incidence of vascular disease and diabetes: an overview. Lipids 45: 925–939. 20397059 10.1007/s11745-010-3412-5 PMC2950929

[6] Ludwig DS, Willett WC (2013) Three daily servings of reduced-fat milk: an evidence-based recommendation? JAMA Pediatr doi:10.1001/jamapediatrics.2013.2408. Accessed 3 July 2013. 10.1001/jamapediatrics.2013.2408 23818041

[7] Rosell M, Hakansson NN, Wolk A (2006) Association between dairy food consumption and weight change over 9 y in 19,352 perimenopausal women. Am J Clin Nutr 84: 1481–1488. 17158433 10.1093/ajcn/84.6.1481

[8] Stancliffe RA, Thorpe T, Zemel MB (2011) Dairy attenuates oxidative and inflammatory stress in metabolic syndrome. Am J Clin Nutr 94: 422–30. 21715516 10.3945/ajcn.111.013342 PMC3142721

[9] Bonthuis M, Hughes MCB, Ibiebele TI, Green AC, van der Pols JC (2010) Dairy consumption and patterns of mortality of Australian adults. Eur J Clin Nutr 64: 569–577. 20372173 10.1038/ejcn.2010.45

[10] Larsson SC, Bergkvist L, Wolk A (2005) High-fat dairy food and conjugated linoleic acid intakes in relation to colorectal cancer incidence in the Swedish Mammography Cohort. Am J Clin Nutr 82: 894–900. 16210722 10.1093/ajcn/82.4.894

[11] Rice BH, Quann EE, Miller GD (2013) Meeting and exceeding dairy recommendations: effects of dairy consumption on nutrient intakes and risk of chronic disease. Nutr Rev 71: 209–223. 23550782 10.1111/nure.12007 PMC3644863

[12] Haug A, Høstmark AT, Harstad OM (2007) Bovine milk in human nutrition—a review. Lipids Health Dis 6: 25 doi:10.1186/1476-511X-6-25 Available: http://www.ncbi.nlm.nih.gov/pmc/articles/PMC2039733. Accessed 2013 Aug 10 17894873 10.1186/1476-511X-6-25 PMC2039733

[13] Simopoulos AP (2006) Evolutionary aspects of diet, the omega-6/omega-3 ratio and genetic variation: nutritional implications for chronic diseases. Biomed Pharmacother 60: 502–507. 17045449 10.1016/j.biopha.2006.07.080

[14] Russo GL (2009) Dietary n-6 and n-3 polyunsaturated fatty acids: From biochemistry to clinical implications in cardiovascular prevention. Biochem Pharma 77: 937–946. 10.1016/j.bcp.2008.10.020 19022225

[15] Couvreur S, Hurtaud C, Lopez C, Delaby L, Peyraud JL (2006) The linear relationship between the proportion of fresh grass in the cow diet, milk fatty acid composition, and butter properties. J Dairy Sci 89: 1956–1969. 16702259 10.3168/jds.S0022-0302(06)72263-9

[16] Lock AL, Bauman DE (2004) Modifying milk fat composition of dairy cows to enhance fatty acids beneficial to human health. Lipids 39: 1197–1206. 15736916 10.1007/s11745-004-1348-6

[17] O’Donnell AM, Spatny KP, Vicini JL, Bauman DE (2010) Survey of the fatty acid composition of retail milk differing in label claims based on production management practices. J Dairy Sci 93: 1918–1925. 20412905 10.3168/jds.2009-2799

[18] Butler G, Nielsen JH, Slots T, Seal C, Eyre MD, et al (2008) Fatty acid and fat-soluble antioxidant concentrations in milk from high- and low-input conventional and organic systems: seasonal variation. J Sci Food Agric 88: 1431–1441.

[19] Stergiadis S, Leifert C, Seal CJ, Eyre MD, Nielsen JH, et al (2012) Effect of feeding intensity and milking system on nutritionally relevant milk components in dairy farming systems in North East of England. J Ag Food Chem 60: 7270–7281. 10.1021/jf301053b 22737968

[20] Krogh LM, Nielsen J, Leifert C, Slots T, Hald Kristensen G, et al (2010) Milk quality as affected by feeding regimes in a country with climatic variation. J Dairy Sci 73: 2863–2873. 10.3168/jds.2009-2953 20630203

[21] Butler G, Nielsen JH, Larsen ML, Rehberger B, Stergiadis S, et al (2011) The effect of dairy management and processing on quality characteristics of milk and dairy products. NJAS – Wageningen J Life Sci 58: 97–102.

[22] Butler G, Stergiadis S, Seal C, Eyre M, Leifert C (2011) Fat composition of organic and conventional retail milk in North East England. J Dairy Sci 94: 24–36. 21183013 10.3168/jds.2010-3331

[23] Slots T, Butler G, Leifert C, Kristensen T, Skibsted LH, et al (2009) Potentials to differentiate milk composition by different feeding strategies. J Dairy Sci 92: 2057–2066. 19389964 10.3168/jds.2008-1392

[24] Blasbalg TL, Hibbeln JR, Ramsden CE, Majchrzak SF, Rawlings RR (2011) Changes in consumption of omega-3 and omega-6 fatty acids in the United States during the 20^th^ century. Am J Clin Nutr 93: 950–962. 21367944 10.3945/ajcn.110.006643 PMC3076650

[25] Hibbeln JR, Nieminen LRG, Blasbalg TL, Riggs JA, Lands WEM (2006) Healthy intakes of ω-3 and ω-6 fatty acids: estimations considering worldwide diversity. Am J Clin Nutr 83: 1483S–1493S. 16841858 10.1093/ajcn/83.6.1483S

[26] Kris-Etherton PM, Taylor DS, Yu-Poth S, Huth P, Moriarty K, et al (2000) Polyunsaturated fatty acids in the food chain in the United States. Am J Clin Nutr 71: 179S–188S. 10617969 10.1093/ajcn/71.1.179S

[27] Masters C (1996) Fatty acids and the peroxisome. Mol Cell Biochem 165: 83–93. 8979256 10.1007/BF00229469

[28] Ailhaud G, Massiera F, Alessandri J-M, Guesnet P (2007) Fatty acid composition as an early determinant of childhood obesity. Genes Nutr 2: 39–40. 18850137 10.1007/s12263-007-0017-6 PMC2474903

[29] Gebauer SK, Psota TL, Harris WS, Kris-Etherton PM (2006) n-3 fatty acid dietary recommendations and food sources to achieve essentiality and cardiovascular benefits. Am J Clin Nutr 83: S1526–15355. 10.1093/ajcn/83.6.1526S 16841863

[30] Yamagishi K, Iso H, Date C, Fukui M, Wakai K, et al (2008) Fish, omega-3 polyunsaturated fatty acids, and mortality from cardiovascular diseases in a nationwide community-based cohort of Japanese men and women. J Am Coll Cardio 52: 988–996. 10.1016/j.jacc.2008.06.018 18786479

[31] De Caterina R (2011) n-3 fatty acids in cardiovascular disease. New Eng J Med 364: 2439–2450. 21696310 10.1056/NEJMra1008153

[32] Molkentin J (2009) Authentication of organic milk using δ^13^C and the α-linolenic acid content of milk fat. J Agric Food Chem 57: 785–790. 19132889 10.1021/jf8022029

[33] Rist L, Mueller A, Barthel C, Snijders B, Jansen M, et al (2007) Influence of organic diet on the amount of conjugated linoleic acids in breast milk of lactating women in the Netherlands. Brit J Nutr 97: 735–743. 17349086 10.1017/S0007114507433074

[34] Woods VB, Fearon AM (2009) Dietary sources of unsaturated fatty acids for animals and their transfer into meat, milk and eggs: A review. Livestock Sci 126: 1–20.

[35] Rinehart L, Baier A (2011) Pasture for organic ruminant livestock: understanding and implementing the national organic program (NOP) pasture rule. Agricultural Marketing Service, U.S. Department of Agriculture. Available: http://www.ams.usda.gov/AMSv1.0/getfile?dDocName=STELPRDC5091036.Accessed 2013May 1.

[36] McBride W, Greene C (2009) Characteristics, costs, and issues for organic dairy farming. Economic research report no. 82, Washington, DC: U.S. Department of Agriculture. Available: http://www.ers.usda.gov/publications/err-economic-research-report/err82.aspx#.UZPDOUouffA. Accessed 2013 May 1.

[37] Donahue SMA, Rifas-Shiman SL, Gold DR, Jouni ZE, Gillman MW, et al (2011) Prenatal fatty acid status and child adiposity at age 3 y: results from a US pregnancy cohort. Am J Clin Nutr 93: 780–788. 21310834 10.3945/ajcn.110.005801 PMC3057547

[38] Brostow DP, Odegaard AO, Koh WP, Duval S, Gross MD, et al (2011) Omega-3 fatty acids and incident type 2 diabetes: the Singapore Chinese Health Study. Am J Clin Nutr 94: 520–526. 21593505 10.3945/ajcn.110.009357 PMC3142726

[39] Moon RJ, Harvey NC, Robinson SM, Ntani G, Davies JH, et al (2013) Maternal plasma polyunsaturated fatty acid status in late pregnancy is associated with offspring body composition in childhood. J Clin Endocrinol Metab 98: 299–307. 23162098 10.1210/jc.2012-2482 PMC3604685

[40] Schuchardt JP, Huss M, Strauss-Grabo M, Hahn A (2010) Significance of long-chain polyunsaturated fatty acids (PUFAs) for the development and behavior of children. Eur J Pediatr 169: 149–164. 19672626 10.1007/s00431-009-1035-8

[41] Ryan AS, Astwood JD, Gautier S, Kuratko CN, Nelson EB, et al (2010) Effects of long-chain polyunsaturated fatty acid supplementation on neurodevelopment in childhood: A review of human studies. Prostag Leukotr ESS 82: 305–314. 10.1016/j.plefa.2010.02.007 20188533

[42] Kummeling I, Thijs C, Huber M, van de Vijver LPL, Snijders BEP, et al (2007) Consumption of organic foods and risk of atopic disease during the first 2 years of life in the Netherlands. Brit J Nutr 99: 598–605. 17761012 10.1017/S0007114507815844

[43] Wang L, Folsom AR, Zheng ZJ, Pankow JS, Eckfeldt JH (2003) Plasma fatty acid composition and incidence of diabetes in middle-aged adults. Am J Clin Nutr 78: 91–98. 12816776 10.1093/ajcn/78.1.91

[44] Djousse L, Biggs ML, Lemaitre RN, King IB, Song X, et al (2011) Plasma omega-3 fatty acids and incident diabetes in older adults. Am J Clin Nutr 94: 527–533. 21593500 10.3945/ajcn.111.013334 PMC3142727

[45] Safarinejad MR, Hosseini SY, Dadkhah F, Asgari MA (2009) Relationship of omega-3 and omega-6 fatty acids with semen characteristics, and antioxidant status of seminal plasma: A comparison between fertile and infertile men. Clin Nutr 29: 100–105. 19666200 10.1016/j.clnu.2009.07.008

[46] Yehuda S, Rabinovitz-Shenkar S, Carasso RL (2011) Effects of essential fatty acids in iron deficient and sleep-disturbed attention deficit hyperactivity disorder (ADHD) children. Eur J Clin Nutr 65: 1167–1169. 21587279 10.1038/ejcn.2011.80

[47] Studer M, Briel M, Leimenstroll B, Glass TR, Bucher HC (2005) Effect of different antilipidemic agents and diets on mortality. Arch Int Med 165: 725–730. 15824290 10.1001/archinte.165.7.725

[48] Ailhaud G, Guesnet P (2004) Fatty acid composition of fats is an early determinant of childhood obesity: a short review and opinion. Obes Rev 5: 21–26. 14969504 10.1111/j.1467-789x.2004.00121.x

[49] AOAC International (2012) Official methods of analysis of AOAC International, 19th ed., Gaithersburg, MD, U.S.

[50] McCullough BD, Heiser DA (2008) On the accuracy of statistical procedures in Microsoft Excel 2007. Comput Stat Data An 52: 4570–4578.

[51] Forkman J (2009) Estimator and tests for common coefficients of variation in normal distributions. Commun Stat A-Theor 38: 233–251.

[52] Smit LA, Baylin A, Campos H (2010) Conjugated linoleic acid in adipose tissue and risk of myocardial infraction. Am J Clin Nutr 92: 34–40. 20463040 10.3945/ajcn.2010.29524 PMC2884320

[53] Li G, Barnes D, Butz D, Bjorling D, Cook M (2005) 10t,12c-conjugated linoleic acid inhibits lipopolysaccharide-induced cyclooxygenase expression in vitro and in vivo. J Lipid Res 46: 2134–2142. 16061956 10.1194/jlr.M500064-JLR200

[54] The role of conjugated linoleic acid in human health. Proceedings of a workshop. Winnipeg, Canada, March 13–15, 2003. Am J Clin Nutr 79: 1131S–1220S. 10.1093/ajcn/79.6.1131S 15159245

[55] U.S. Department of Agriculture (2012) National nutrient database for standard reference, release 25. Available: http://ndb.nal.usda.gov. Accessed 2013 Mar 8.

[56] Glasser F, Doreau M, Ferlay A, Chilliard Y (2007) Technical note: estimation of milk fatty acid yield from milk fat data. J Dairy Sci 90: 2302–2304. 17430931 10.3168/jds.2006-870

[57] Rymer C, Givens DI, Wahle KWJ (2003) Dietary strategies for increasing docosahexaenoic acid (DHA) and eicosapentaenoic acid (EPA) concentrations in bovine milk: a review. Nutr Abs Rev Series B, Livestock Feeds and Feeding 73: 9R–25R.

[58] Palupi E, Jayanegara A, Ploegera A, Kahl J (2012) Comparison of nutritional quality between conventional and organic dairy products: a meta-analysis. J Sci Food Agric DOI: 10.1002/jsfa.5639. 10.1002/jsfa.5639 22430502

[59] U.S. Department of Agriculture (2008) Dairy 2007, Part II: Changes in the U.S. dairy cattle industry, 1991–2007, USDA-APHIS-VS, CEAH, Fort Collins, Co. #N481.0308. Available: http://www.aphis.usda.gov/animal_health/nahms/dairy/downloads/dairy07/Dairy07_dr_PartII.pdf. Accessed 2013 May 1.

[60] Mathews KH, McConnell MJ (2009) Ethanol co-product use in U.S. cattle feeding: lessons learned and considerations. FDS-09D-01, Economic Research Service, U.S. Department of Agriculture. Available: http://usda01.library.cornell.edu/usda/ers/FDS//2000s/2009/FDS-04-17-2009_Special_Report.pdf. Accessed 2013 Oct 10.

[61] Butler LJ (2002) The economics of organic milk production in California: a comparison with conventional costs. Am J Alt Agric 17: 83–91.

[62] Massiera F, Barbry P, Guesnet P, Joly A, Luquet S, et al (2010) A Western-like fat diet is sufficient to induce a gradual enhancement in fat mass over generations. J Lipid Res 51: 2352–2361. 20410018 10.1194/jlr.M006866 PMC2903802

[63] Meyer BJ, Mann NJ, Lewis JL, Milligan GC, Sinclair AJ, et al (2006) Dietary intakes and food sources of omega-6 and omega-3 polyunsaturated fatty acids. Lipids 38: 391–398. 10.1007/s11745-003-1074-0 12848284

[64] Wood JAT, Williams JS, Pandarinathan L, Janero DR, Lammi-Keefe CJ, et al (2010) Dietary docosahexaenoic acid supplementation alters select physiological endocannabinoid-system metabolites in brain and plasma. J Lipid Res 51: 1416–1423. 20071693 10.1194/jlr.M002436 PMC3035504

[65] Amminger GP, Schafer MR, Papageorgiou K, Klier CM, Cotton SM, et al (2010) Long-chain ω-3 fatty acids for indicated prevention of psychotic disorders: a randomized, placebo-controlled trial. Arch Gen Psychiatry 67: 146–154. 20124114 10.1001/archgenpsychiatry.2009.192

[66] Ruxton CHS, Calder PC, Reed SC, Simpson MJA (2005) The impact of long-chain n-3 polyunsaturated fatty acids on human health. Nutr Res Rev 18: 113–129. 19079899 10.1079/NRR200497

[67] Childs CE, Romeu-Nadal M, Burdge GC, Calder PC (2008) Gender differences in the ω-3 fatty acid content of tissues, Proc Nutr Soc. 67: 19–27. 10.1017/S0029665108005983 18234128

[68] Pawlosky R, Hibbeln RJ, Lin Y, Salem N (2003) n-3 fatty acid metabolism in women. Brit J Nutr 90: 993–994. 14667193 10.1079/bjn2003985

[69] Williams CM, Burdge G (2006) Long-chain n-3 PUFA: plant v. marine sources. Proc Nutr Soc 65: 42–50. 16441943 10.1079/pns2005473

[70] Gibson RA, Muhlhausler B, Makrides M (2011) Conversion of linoleic acid and alpha-linolenic acid to long-chain polyunsaturated fatty acids (LCPUFAs), with a focus on pregnancy, lactation and the first 2 years of life. Matern Child Nutr 7 Suppl 217–26. 21366864 10.1111/j.1740-8709.2011.00299.x PMC6860743

[71] Emkin EA, Adlof RO, Gulley RM (1994) Dietary linoleic acid influences desaturation and acylation of deuterium-labeled linoleic and linolenic acids in young adult males. Biochim Biophys Acta 1213: 277–288. 7914092 10.1016/0005-2760(94)00054-9

[72] Arterburn LM, Hall EB, Oken H (2006) Distribution, interconversion, and dose response of n-3 fatty acids in humans. Am J Clin Nutr 83: 1467S–1476S. 16841856 10.1093/ajcn/83.6.1467S

[73] Burdge GC, Calder PC (2005) α-Linolenic acid metabolism in adult humans: the effects of gender and age on conversion to longer-chain polyunsaturated fatty acids. Eur J Lipid Sci Technol 107: 426–439.

[74] Brenna JT (2002) Efficiency of conversion of alpha-linolenic acid to long-chain n-3 fatty acids in man. Curr Opin Clin Nutr Metab Care 5: 127–132. 11844977 10.1097/00075197-200203000-00002

[75] Burdge GC, Calder PC (2005) Conversion of alpha-linolenic acid to longer-chain polyunsaturated fatty acids in human adults. Reprod Nutr Dev 45: 581–597. 16188209 10.1051/rnd:2005047

[76] Gebauer SK, Chardigny J-M, Jakobsen MU, Lamarche B, Lock AL, et al (2011) Effects of ruminant *trans* fatty acids on cardiovascular disease and cancer: a comprehensive review of epidemiological, clinical, and mechanistic studies. Adv Nutr 2: 332–354. 22332075 10.3945/an.111.000521 PMC3125683

[77] U.S. Food and Drug Administration (2003) Regulation 21 CFR 101.9(c)(2)(ii) “*Trans* fat” or “*Trans*” Federal Register 68: 41502. Available: http://web.archive.org/web/20070103035701/http://www.cfsan.fda.gov/~acrobat/fr03711a.pdf. Accessed 2013 May 15.

[78] Lacroix E, Charest A, Cyr A, Baril-Gravel L, Lebeuf Y, et al (2012) Randomized controlled study of the effect of a butter naturally enriched in trans fatty acids on blood lipids in healthy women. Am J Clin Nutr 95: 318–325. 22205319 10.3945/ajcn.111.023408 PMC3260067

