# Supplementary figures and images for: Organic Production Enhances Milk Nutritional Quality by Shifting Fatty Acid Composition: A United States–Wide, 18-Month Study
Source: PLoS One. 2013 Dec 9;8(12):e82429. doi: 10.1371/journal.pone.0082429 (PMC3857247; doi:10.1371/journal.pone.0082429)

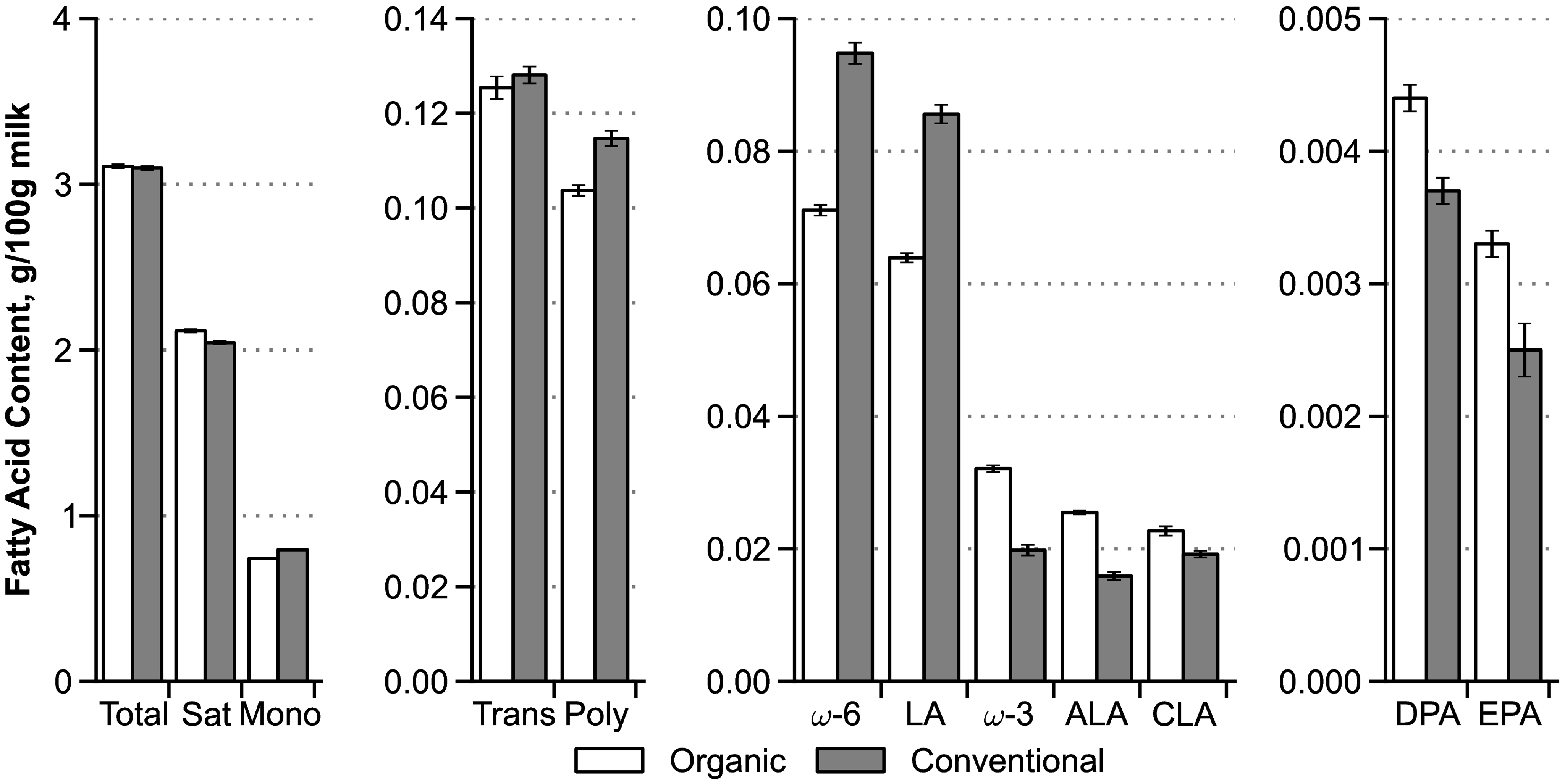

Supplement: Figure S1 — Fatty acid content of retail whole milk, g/100 g (12-month average ± SE). Some SE are too small to be visible. Abbreviations: Sat = saturated, Mono = monounsaturated, Poly = polyunsaturated, LA = linoleic acid, ALA = α-linolenic acid, CLA = conjugated linoleic acid, EPA = eicosapentaenoic acid, DPA = docosapentaenoic acid. Differences between organic and conventional contents are statistically significant by Mann-Whitney test (P<0.001) except for Total and Trans fatty acids (P > 0.40). (TIF) [file pone.0082429.s001.tif]
