# Supplementary material for: Organic Production Enhances Milk Nutritional Quality by Shifting Fatty Acid Composition: A United States–Wide, 18-Month Study
Source: PLoS One. 2013 Dec 9;8(12):e82429. doi: 10.1371/journal.pone.0082429 (PMC3857247; doi:10.1371/journal.pone.0082429)
Supplement: Table S1 — (DOC) [file pone.0082429.s003.doc]

**Table S1.** Fatty acids as percent of total fatty acids in retail whole milk, 12 months ending Dec. 2011.

|  |  | **Organic** | | | | | **Conventional** | | | | | **Org/** | ***P*(differ-** |
| --- | --- | --- | --- | --- | --- | --- | --- | --- | --- | --- | --- | --- | --- |
|  |  | **Mean** | **n** | **SD** | **CV** | **SE** | **Mean** | **n** | **SD** | **CV** | **SE** | **Conv** | **ence)*** |
| **Saturated fatty acids** | | | | | | | | | | | | | |
|  | 4:0 butyric | 2.418 | 143 | 0.178 | 7.4% | 0.015 | 2.383 | 107 | 0.189 | 7.9% | 0.018 | 1.01 | 0.14 |
|  | 6:0 caproic | 1.952 | 143 | 0.185 | 9.5% | 0.015 | 1.861 | 108 | 0.167 | 9.0% | 0.016 | 1.05 | 0.00008 |
|  | 8:0 caprylic | 1.308 | 143 | 0.141 | 11% | 0.012 | 1.238 | 108 | 0.103 | 8.4% | 0.010 | 1.06 | 0.00002 |
|  | 10:0 capric | 4.140 | 143 | 0.978 | 24% | 0.082 | 4.004 | 108 | 0.891 | 22% | 0.086 | 1.03 | 0.26 |
|  | 11:0 undecylic | 0.083 | 85 | 0.031 | 37% | 0.003 | 0.085 | 84 | 0.028 | 33% | 0.003 | 0.97 | 0.64 |
|  | 12:0 lauric | 3.405 | 143 | 0.371 | 11% | 0.031 | 3.194 | 108 | 0.297 | 9.3% | 0.029 | 1.07 | 0.00000 |
|  | 14:0 myristic | 11.23 | 143 | 0.617 | 5.5% | 0.052 | 10.57 | 108 | 0.670 | 6.3% | 0.064 | 1.06 | 0.00000 |
|  | 15:0 pentadecanoic | 1.294 | 143 | 0.108 | 8.4% | 0.009 | 1.144 | 108 | 0.115 | 10% | 0.011 | 1.13 | 0.00000 |
|  | 16:0 palmitic | 30.06 | 143 | 2.140 | 7.1% | 0.179 | 29.03 | 108 | 1.249 | 4.3% | 0.120 | 1.04 | 0.00001 |
|  | 17:0 margaric | 0.781 | 143 | 0.066 | 8.4% | 0.005 | 0.696 | 108 | 0.075 | 11% | 0.007 | 1.12 | 0.00000 |
|  | 18:0 stearic | 11.05 | 143 | 1.197 | 11% | 0.100 | 11.49 | 108 | 1.086 | 9.5% | 0.105 | 0.96 | 0.0032 |
|  | 20:0 arachidic | 0.207 | 141 | 0.043 | 21% | 0.004 | 0.178 | 107 | 0.031 | 18% | 0.003 | 1.16 | 0.00000 |
|  | 22:0 behenic | 0.130 | 105 | 0.030 | 23% | 0.003 | 0.104 | 61 | 0.041 | 40% | 0.005 | 1.25 | 0.00001 |
|  | 24:0 lignoceric | 0.079 | 73 | 0.031 | 40% | 0.004 | 0.076 | 23 | 0.031 | 40% | 0.006 | 1.04 | 0.69 |
|  | Total saturated† | 68.06 | 143 | 2.092 | 3.1% | 0.175 | 65.94 | 108 | 1.682 | 2.6% | 0.162 | 1.03 | 0.00000 |
| **Monounsaturated fatty acids** | | | | | | | | | | | | | |
|  | 14:1 myristoleic | 0.933 | 143 | 0.111 | 12% | 0.009 | 0.868 | 108 | 0.115 | 13% | 0.011 | 1.07 | 0.00001 |
|  | 16:1 palmitoleic | 1.506 | 143 | 0.192 | 13% | 0.016 | 1.506 | 108 | 0.216 | 14% | 0.021 | 1.00 | 0.99 |
|  | 17:1 margaroleic | 0.257 | 137 | 0.044 | 17% | 0.004 | 0.227 | 106 | 0.046 | 20% | 0.004 | 1.13 | 0.00000 |
|  | 18:1 incl. oleic | 20.93 | 143 | 1.391 | 6.6% | 0.116 | 22.84 | 108 | 1.357 | 5.9% | 0.131 | 0.92 | 0.00000 |
|  | 20:1 incl. gadoleic | 0.227 | 126 | 0.083 | 37% | 0.007 | 0.217 | 96 | 0.082 | 38% | 0.008 | 1.05 | 0.37 |
|  | Total monounsaturated† | 23.84 | 143 | 1.294 | 5.4% | 0.108 | 25.64 | 108 | 1.289 | 5.0% | 0.124 | 0.93 | 0.00000 |
| ω**-3 fatty acids** | | | | | | | | | | | | | |
|  | 18:3 α-linolenic, ALA | 0.820 | 143 | 0.128 | 16% | 0.011 | 0.511 | 108 | 0.185 | 36% | 0.018 | 1.61 | 0.00000 |
|  | 20:5 eicosapentaenoic, EPA | 0.105 | 104 | 0.037 | 35% | 0.004 | 0.079 | 43 | 0.032 | 41% | 0.005 | 1.33 | 0.00009 |
|  | 22:5 docosapentaenoic, DPA | 0.141 | 120 | 0.040 | 28% | 0.004 | 0.119 | 70 | 0.030 | 25% | 0.004 | 1.19 | 0.00007 |
|  | Total ω-3† | 1.032 | 143 | 0.190 | 18% | 0.016 | 0.635 | 108 | 0.266 | 42% | 0.026 | 1.62 | 0.00000 |
| ω**-6 fatty acids** | | | | | | | | | | | | | |
|  | 18:2 linoleic, LA | 2.057 | 143 | 0.240 | 12% | 0.020 | 2.765 | 107 | 0.458 | 17% | 0.044 | 0.74 | 0.00000 |
|  | 20:3 8,11,14-eicosatrienoic (γ) | 0.104 | 110 | 0.033 | 31% | 0.003 | 0.139 | 92 | 0.037 | 27% | 0.004 | 0.75 | 0.00000 |
|  | 20:4 arachidonic, AA | 0.155 | 118 | 0.046 | 30% | 0.004 | 0.185 | 91 | 0.053 | 28% | 0.006 | 0.84 | 0.00001 |
|  | Total ω-6† | 2.286 | 143 | 0.282 | 12% | 0.024 | 3.060 | 107 | 0.515 | 17% | 0.050 | 0.75 | 0.00000 |
|  | Total Polyunsaturated† | 3.336 | 143 | 0.382 | 11% | 0.032 | 3.701 | 107 | 0.493 | 13% | 0.048 | 0.90 | 0.00000 |
| ***trans* fatty acids** | | | | | | | | | | | | | |
|  | *trans*-16:1 *trans*-palmitoleic | 0.420 | 143 | 0.069 | 16% | 0.006 | 0.378 | 108 | 0.060 | 16% | 0.006 | 1.11 | 0.00000 |
|  | *trans*-18:1 incl. elaidic | 2.724 | 143 | 0.762 | 28% | 0.064 | 2.923 | 108 | 0.432 | 15% | 0.042 | 0.93 | 0.00004‡ |
|  | *trans*-18:2 octadecadienoic | 0.822 | 143 | 0.257 | 31% | 0.022 | 0.782 | 107 | 0.183 | 23% | 0.018 | 1.05 | 0.17 |
|  | Total *trans*§ | 4.032 | 143 | 0.917 | 23% | 0.077 | 4.128 | 108 | 0.542 | 13% | 0.052 | 0.98 | 0.33 |
| **Conjugated linoleic acid, CLA** | | | | | | | | | | | | | |
|  | 18:2 conjugated | 0.730 | 143 | 0.270 | 37% | 0.023 | 0.619 | 106 | 0.150 | 24% | 0.015 | 1.18 | 0.00017 |
| **Sum** | | | | | | | | | | | | | |
|  | ALA + CLA | 1.550 | 143 | 0.335 | 22% | 0.028 | 1.118 | 108 | 0.300 | 27% | 0.029 | 1.39 | 0.00000 |
| **Ratios** | | | | | | | | | | | | | |
|  | LA/ALA | 2.568 | 143 | 0.544 | 21% | 0.046 | 6.272 | 107 | 2.485 | 40% | 0.240 | 0.41 | 0.00000 |
|  | ω-6/ω-3 | 2.276 | 143 | 0.469 | 21% | 0.039 | 5.774 | 107 | 2.520 | 44% | 0.244 | 0.39 | 0.00000 |
|  | ω-3/ω-6 | 0.456 | 143 | 0.083 | 18% | 0.007 | 0.219 | 107 | 0.124 | 57% | 0.012 | 2.08 | 0.00000 |
|  | ω-6/(ω-3 + CLA) | 1.353 | 143 | 0.345 | 26% | 0.029 | 2.742 | 107 | 1.223 | 45% | 0.122 | 0.49 | 0.00000 |

* Calculated by *t* test except as indicated. Because of multiple comparisons, about 2 findings of *P* = 0.05 and 0.5 finding of *P* = 0.01 can be expected by chance in this table

† These group means (means of sums of saturated, monounsaturated, or polyunsaturated FA) are biased slightly low, because they include some sums containing unreported small values (< 0.001) treated as zero. In contrast, when n is less than the number of samples (organic n < 143, conventional n < 108), means of individual FA are biased slightly high by omission of unreported small values. Thus these group means are slightly less than the sum of means of the individual FA.

‡ Calculated by Mann-Whitney test due to non-normal distributions with medians 2,77 (organic) and 2.59 (conventional). (*P* = 0.016 calculated by *t* test).

§ The *trans* FA group mean exceeds the sum of individual *trans* FA, because it includes small amounts of *trans*-14:1 omitted from the table due to small numbers of reported values (42 organic, 27 conventional).
